# Amino acid composition in eyes from zebrafish (*Danio rerio*) and sardine (*Sardina pilchardus*) at the larval stage

**DOI:** 10.1186/s40064-016-2137-1

**Published:** 2016-04-26

**Authors:** Francesca Falco, Marco Barra, Matteo Cammarata, Angela Cuttitta, Sichao Jia, Angelo Bonanno, Salvatore Mazzola, Guoyao Wu

**Affiliations:** Detached Units of Capo Granitola (TP) and Naples, Institute for Coastal and Marine Environment (IAMC), Consiglio Nazionale delle Ricerche, Capo Granitola (TP), Italy; Department of Animal Science, Texas A&M University, College Station, TX 77843 USA; Marine Immunobiology Laboratory, Department of Biological, Chemical, Pharmaceutical Science and Technology, University of Palermo, Palermo, Italy

**Keywords:** Larval fish, Eye, Amino acid composition, *Danio rerio*, *Sardina pilchardus*

## Abstract

A comparative study was performed to identify differences in the amino acid composition of the eyes from zebrafish (*Danio rerio*) and sardine (*Sardina pilchardus*) larvae and their link to the environmental adaption of the species. Amino acids in the acidic hydrolysates of eyes from 11 zebrafish and 12 sardine were determined with the use of high-performance liquid chromatography involving precolumn derivatization with ortho-phthalaldehyde. Differences in the content of most amino acids were detected between zebrafish and sardine. These amino acids were aspartate, glutamate, serine, glycine, threonine, arginine, methionine, valine, phenylalanine, isoleucine, leucine and lysine. Of particular note, the percentage of methionine in zebrafish eyes was much higher than that in sardine, whereas the opposite was observed for glutamate and glycine. These results indicate that zebrafish and sardine likely have experienced differences in adaptation to environmental changes. We suggest that the amino acid composition of eyes represents a powerful tool to discriminate between species characterized by different lifestyle and inhabiting different environments.

## Background

Recent studies led to the discovery that the genes involved in the eye ontogeny are conserved and that all of the eyes are monophyletic, that is, they arose from a single eye origin (Gehring and Ikeo 1999; Russell et al. 2000; Fernald 2000). Likewise, the conservation of a specific transcription factor has a common evolutionary origin for all eyes (Treisman 2004), and regulatory effect of certain AA on gene expression may be mediated by transcription factors (Wu 2010, 2013a).

Salvini-Plawen and Mayr (1977) demonstrated that the eye evolved at least 40 times among the branches of the animal evolutionary tree. The evolutionary phenomenon that led to the development of complex eyes, as those of mammals from teleost fish, is due to different factors, such as the influence of specific environmental changes on the biochemical composition of the tissue structure of a living being (Brown and Taylor 1992; Nissling and Vallin 1996; Guisande et al. 1998; Riveiro et al. 2000, 2003).

According to the Darwinist idea that animals adapted to the environment where they live in order to survive to specific environmental pressures, changes in amino acid composition (AAC) and density patterns of pelagic and mesopelagic fish larvae were evidenced in relation to oceanographic phenomenon in different areas of the Central Mediterranean Sea (Cuttitta et al. 2004, 2006; Bonanno et al. 2013). Another factor that could affect the biochemical composition of the tissues is that their synthesis during the larval development happens at different times and rates (Osse et al. 1997).

Conceição et al. (1998) showed that in the larval catfish (*Clarias gariepinus*) changes in the amino acid profiles occur at different temperatures, due to the synthesis of additional proteins during larval growth. Such environmental influence on biochemical composition lead to of use of AAC in eggs and larvae of fish to discriminate among species and spawning areas within species (Riveiro et al. 2003), considering also that larval AAC in pelagic fishes may also be affected by the parental strategies (Baynes and Howell 1996; Riveiro et al. 2011).

According to the preliminary study of Riveiro et al. (2011), the eyes may be the best fish tissue to discriminate among species through the AAC analysis. Generally all cells have a basal requirement for amino acids in processes such as protein synthesis (Wu 2013a). Amino acids are building blocks of proteins and also regulate metabolic processes in the body (Hou et al. 2015; Wu 2013b).

Amino acids play a critical role also in healthy vision. Interestingly, the most abundant amino acids in vertebrate’s eyes are glutamate (for gamma amino-butyric acid synthesis; GABA) and GLY (Neal 1976; Massey and Redburn 1987; Massey and Miller 1988; Barnstable 1993; Pourcho 1996; Redburn 1998; Thoreson and Witkovsky 1999). In addition to peptide-bound amino acids, eyes also contain free amino acids, including aspartate, asparagine, glutamate, glutamine, glycine, serine, proline, homocysteine, and taurine. At present, little is known about the ocular content of total amino acids in eyes. In addition, reference values of AAC in the eyes of larval fish of different species are not available in literature.

In this study we focused our attention on two fish species at larval stage: the cyprinidae zebrafish (*Danio rerio*), a tropical freshwater species, and sardine (*Sardina pilchardus*), belonging to the Clupeidae family that is a typical pelagic species living in open sea waters. The two species differ markedly in biology, habitat, and growth rates. The results can be used to assess the adaptation of the species to different environmental conditions.

## Results

**Fig. 1:**
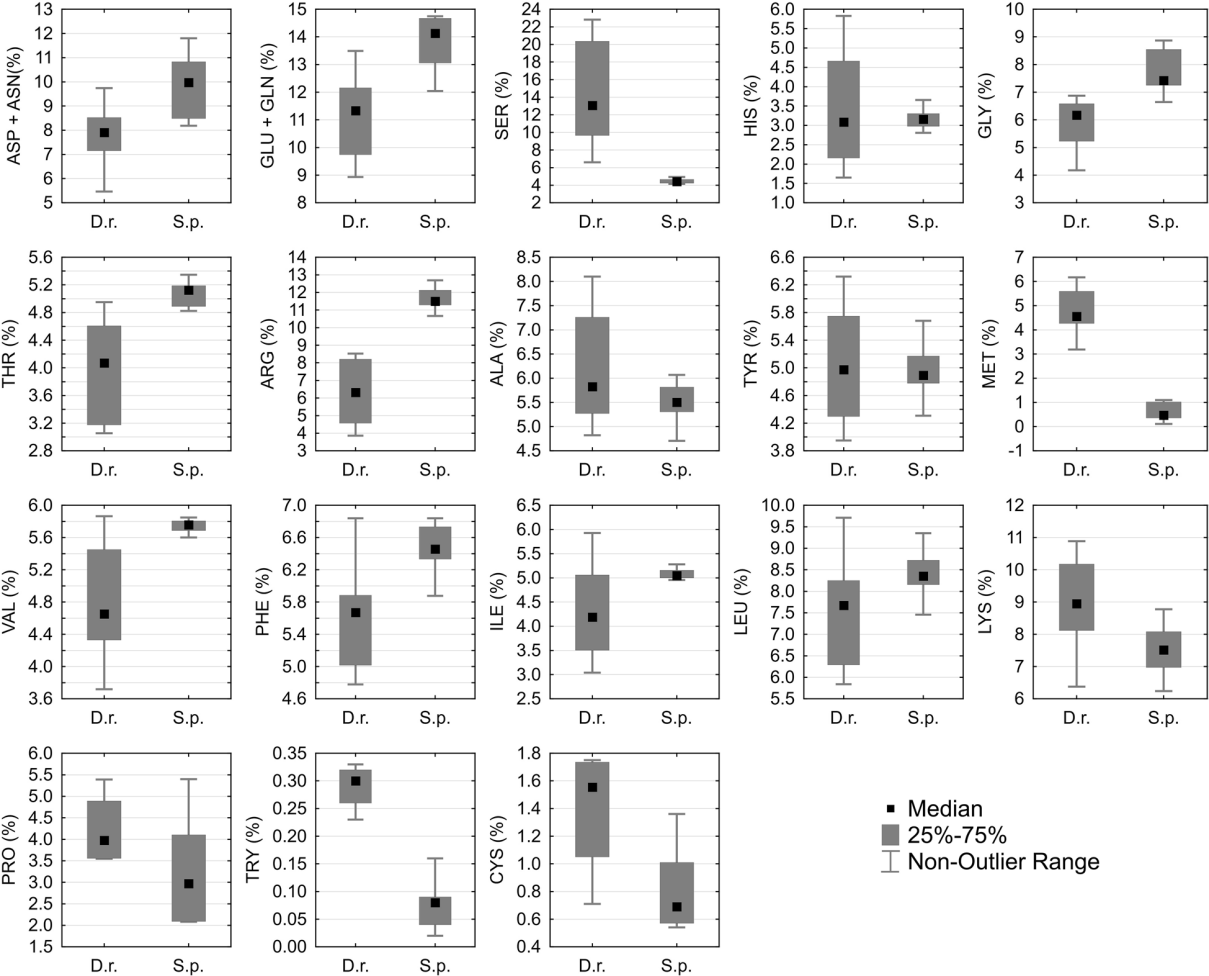
*Box plot* comparing amino acid composition (g/100 g amino acids; %) between zebrafish (*Danio rerio*) and sardine (*Sardina pilchardus*) larvae

We analyzed the total content of amino acids (both free and peptide-bound) in the eyes of two fish species: zebrafish (*Danio rerio*) and sardine (*Sardina pilchardus*) at the larval stage. The mean and median values on amino acid composition (g/100 g amino acids) in the eyes of the two fish species are reported in Table 1. Reported values show that both mean and median percentage compositions (Fig. 1) of amino acids in the eyes of *S. pilchardus* are generally higher (p < 0.05) than those ones in *D. rerio*, especially for ASP + ASN, GLU + GLN, GLY, THR, ARG, PHE, VAL, and LEU. On the contraty, mean and median percentage compositions of SER, MET and LYS in the eyes of *D. rerio* were higher than those in *S. pilchardus.* Furthermore, except for GLY and ASP + ASN, it was evident that AAC percentage values showed generally higher variability (as inferred by interquartile range—Fig. 1) in *D. rerio* than in *S. pilchardus*.

**Table 1:** Median and mean values of the composition of amino acids in the eyes of *Danio rerio* and *Sardina pilchardus* larvae

Amino acid (g/100 g amino acids; %)	*Danio rerio* (*n* = 11)				
	Median	Quartile range	Min–max	Mean	SE
ASP + ASN	7.9	1.4	5.5–9.7	7.80	0.32
GLU + GLN	11.3	2.4	8.9–13.5	11.16	0.46
SER	13.1	10.7	6.6–22.8	14.04	1.66
HIS	3.1	2.5	1.7–5.8	3.31	0.41
GLY	6.2	1.4	4.2–9.4	6.15	0.40
THR	4.1	1.4	3.1–5	4.02	0.21
ARG	6.3	3.6	3.9–8.5	6.39	0.51
ALA	5.8	2.0	4.8–8.1	6.19	0.35
TYR	5.0	1.5	4–6.3	5.03	0.24
MET	4.6	1.3	2.2–6.2	4.65	0.36
VAL	4.7	1.1	3.7–5.9	4.76	0.22
PHE	5.7	0.9	4.8–6.8	5.63	0.20
ILE	4.2	1.6	3.0–5.9	4.29	0.28
LEU	7.7	2.0	5.8–9.7	7.52	0.39
LYS	8.9	2.1	6.4–10.9	9.07	0.42
CYS	1.6	0.7	0.7–1.8	1.39	0.24
TRP	0.3	0.1	0.2–0.3	0.29	0.02
PRO	4.0	1.3	3.6–5.4	4.22	0.44

Amino acid (%)	*Sardina pilchardus* (*n* = 12)				
	Median	Quartile range	Min–max	Mean	SE
ASP + ASN	10	2.3	2.7–11.8	9.34	0.69
GLU + GLN	14.1	1.6	6.2–14.7	13.33	0.70
SER	4.4	0.4	4.1–4.9	4.46	0.08
HIS	3.2	0.3	1.1–3.7	3.02	0.19
GLY	7.4	1.3	6.6–11.6	7.95	0.39
THR	5.1	0.3	4.3–5.3	5.03	0.08
ARG	11.5	0.8	10.7–15.9	11.96	0.39
ALA	5.5	0.5	4.7–6.1	5.51	0.11
TYR	4.9	0.4	4.3–5.7	4.97	0.11
MET	0.5	0.7	0.1–2.3	0.80	0.21
VAL	5.8	0.1	5.6–6	5.76	0.03
PHE	6.5	0.4	5.9–8	6.59	0.15
ILE	5.0	0.2	5.0–5.3	5.08	0.03
LEU	8.4	0.6	7.5–9.8	8.49	0.18
LYS	7.5	1.1	6.2–11	7.72	0.37
CYS	0.7	0.4	0.5–1.4	0.83	0.16
TRP	0.1	0.1	0.0–0.2	0.08	0.02
PRO	3.0	2	2.1–5.4	3.33	0.64

Statistical tests were carried out in order to evaluate the significance of observed differences in terms of AAC (Table 2). In particular, the Mann–Whitney U test was used because not all the data met homoscedasticity assumptions, as required by parametric tests. Indeed, the Levene’s test (not shown) evidenced a significant difference in variance between the two groups of fish for some amino acids. Test results (Table 2) highlighted that median values of HIS, ALA and TYR were not significantly different (p > 0.05) between the two considered species. Conversely, significant differences were recorded for all the other AACs (p < 0.05). In particular, SER, ARG and MET showed the highest differences in median values with respect to the other AACs (Table 2).

**Table 2:** Mann Whitney U test results for differences in amino acid composition in eyes between *Danio Rerio* and *Sardina pilchardus* larvae

Amino acid	U	Z	Z adj.	p-value	Median differences
ASP + ASN	19	−*2.862*	−*2.862*	*0.004*	2.1
GLU + GLN	17	−*2.985*	−*2.985*	*0.003*	2.8
SER	0	*4.031*	*4.031*	*0.000*	8.7
HIS	61	−0.277	−0.277	0.782	0.1
GLY	12	−*3.293*	−*3.293*	*0.001*	1.3
THR	8	−*3.539*	−*3.539*	*0.000*	1.1
ARG	0	−*4.031*	−*4.031*	*0.000*	5.2
ALA	50	0.954	0.954	0.340	0.3
TYR	61	0.277	0.277	0.782	0.1
MET	2	*3.908*	*3.908*	*0.000*	4.1
VAL	20	−*2.800*	−*2.800*	*0.005*	1.1
PHE	19	−*2.862*	−*2.862*	*0.004*	0.8
ILE	31	−*2.123*	−*2.123*	*0.034*	0.9
LEU	28	−*2.308*	−*2.308*	*0.021*	0.7
LYS	29	*2.246*	*2.246*	*0.025*	1.4
CYS	2	1.837	1.837	0.066	0.9
PRO	6	0.857	0.857	0.391	1.0
TRP	0	*2.327*	*2.327*	*0.02*	0.2

Significant differences are marked in italic. The absolute differences in median values were also reported

**Fig. 2:**
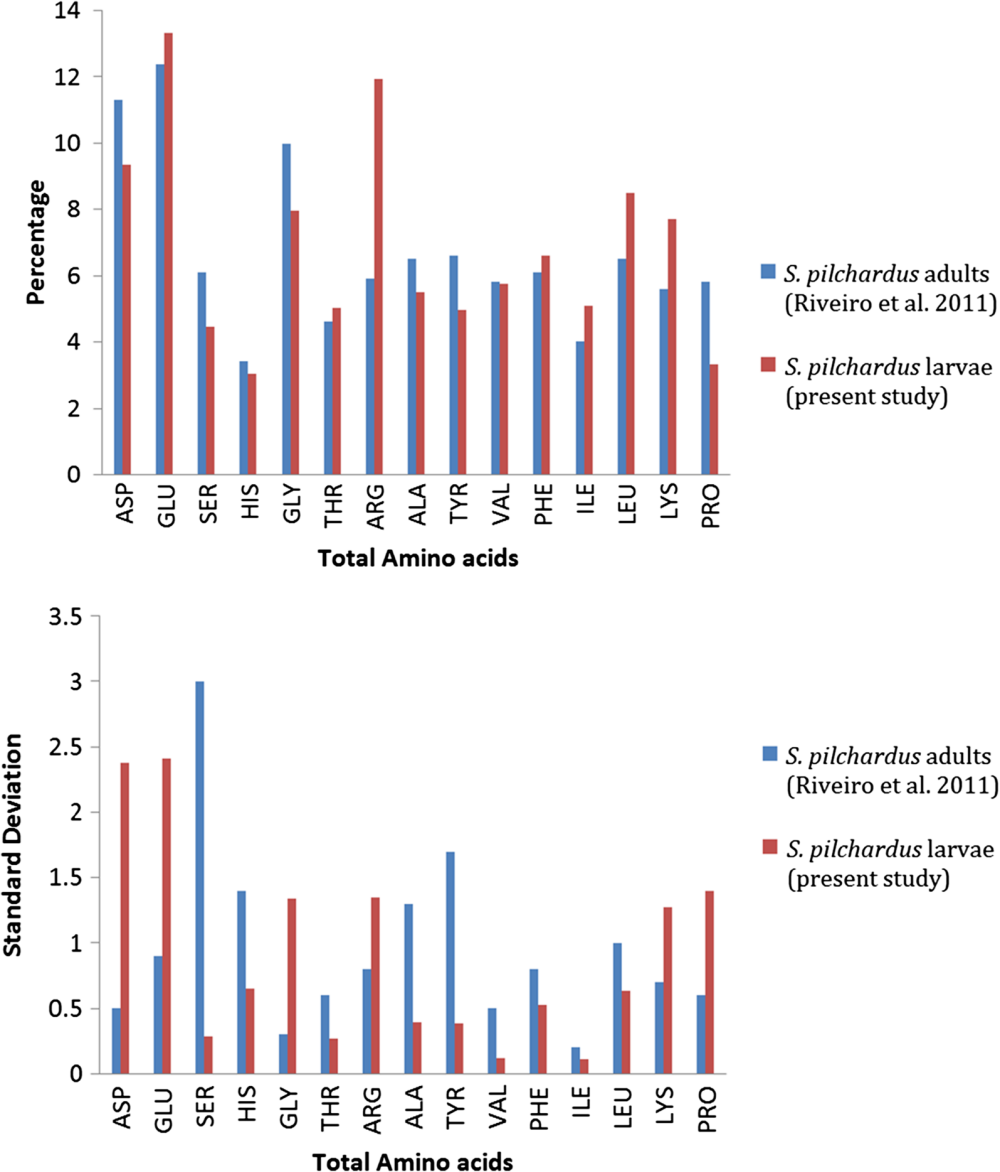
Comparison of the average amino acid composition (*top panel*) and SD values (*bottom panel*) between adults (Riveiro et al. 2011) and larvae (this study) of the species *Sardina pilchardus*

The mean and standard deviation values for *S. pilchardus* were compared with those obtained on the amino acid content of the eyes of adult fish of the same species (Fig. 2) obtained from the Strait of Sicily (Riveiro et al. 2011). Our results showed higher standard deviations for ASP, GLY, GLU, ARG, LYS and PRO, compared to the values reported by Riveiro et al. (2011); the opposite was observed for the remaining AAs.

## Discussion

Results of the present study provide reference values of amino acid content in the eyes of zebrafish and sardine for the larval stage. As free amino acids represent <3 % of total amino acids in tissues (Wu 2013a), our values refer to primarily peptide-bound amino acids in the eyes of the fish. The amino acid composition found in this work could be compared with the amino acid composition in structural proteins of the retina (Harding and Dilley 1976; Wistow and Piatigorsky 1988; Zhao et al. 2011). Our results showed that it is possible to discriminate fish species based on the AAC of the eyes. Among the considered AA, the two species showed marked differences particularly in SER, ARG and MET. It is unknown whether the differences in AAC of the fish eyes result from differences in dietary protein intake and/or plasma concentrations of amino acids. It has been demonstrated that SER, ARG and MET have a greater insulinotropic effect compared with glucose in fish (Andoh 2007; Zinalla and Hall 2008). Further, MET has growth-promoting effects in the rainbow trout (Rodehutscord et al. 1995).

In the eyes of larval zebrafish, the amount of methionine was lower than in the sardine larvae but the opposite was observed for arginine. The difference between the species was smaller for lysine. Moreover, it was found that GLU + GLN and GLY were higher in sardine than in zebrafish. Whether these differences are unique to the eyes or common to other fish tissues remain to be determined.

The mean and standard deviation values for *S. pilchardus* were compared with those for the amino acid concentration of the eyes of adult fish of the same species obtained from the Sicilian Channel. The composition of most amino acids in the eyes of adult and larval specimens of *S. pilchardus* was similar (Fig. 2); only ASP, SER, GLY, ARG, LEU and LYS appeared to have quite different values. On the basis of this result, we surmise that ASP, SER and GLY are generally more abundant in adults, while ARG, LEU and LYS were higher in larvae than in adults. Such differences within the same species, during growth from the larval to adult stage, are in agreement with the findings of Conceição et al. (1997, 2010) and Aragão et al. (2004) who carried out the study of the AAC in the whole body of larval fish.

In particular, the observed higher glycine concentration in adults than in the sardine larvae is in agreement with Sivilotti (2010), which highlights the synaptic role of GLY. It is possible that the eyes of adult fish have higher content of collagen proteins than larvae, because glycine is a major amino acid in these proteins (Wang et al. 2013). The glicinergic synapses are important in restricted areas of the adult nervous system, such as the spinal cord, brain stem and retina. They are activated primarily by GLY, but can also be activated by common amino acids.

The ASP was classified by Wu (2013b) as a conditionally essential AA. Abundant AA in food proteins of plant and animal origins (Li et al. 2011) is a major metabolic fuel for mammalian enterocytes (Burrin and Stoll 2009; Rezaei et al. 2013a, b). Further, Wu (2010) and Wu (2013b) found that some Amino Acids are involved in regulating the metabolic key pathways improving health, survival, growth, development, lactation, and reproduction of organisms. At present, little is known about ASP metabolism in fish. According to Kim et al. (2011a, b), Wu et al. (2011a, b) and Wu (2013b), the GLY, together with other amino acids, was traditionally classified as non-essential amino acids, but these amino acids play an important role in regulating gene expression (Liu et al. 2012), cell signaling (Bazer et al. 2012; Jewell et al. 2013), nutrient transport and metabolism in animal cells (Suryawan et al. 2012; Wang et al. 2013). Regarding SER and LYS, their high concentration has been reported in the whole body of fish at the larval period (Zakeri et al. 2009). As building blocks of peptides, these amino acids have an important role in the synthesis of protein. Like many of the other amino acids (Li et al. 2007a), SER and LYS may be critical for immune response during the larval stage.

According to Kalloniatis et al. (2013), amino acids are also involved in metabolism, and in retina cell, glutamate is the major excitatory neurotransmitter in the retina (Fletcher and Kalloniatis 1996; Ehinger et al. 1988; Massey and Miller 1990). Glutamate is also the precursor of GABA (Erecinska and Silver 1990) and there is strong evidence that glutamate is used by photoreceptors (Massey and Miller 1990, 1998).

Riveiro et al. (2011) found that there were differences in the AAC of the eyes of adult sardine between samples from two different regions (the Atlantic Ocean and the Mediterranean Sea). With the available data, it is not possible to determine whether the different environmental conditions may affect AAC in the eyes of zebrafish and sardine reported from the present study. However, this is a very important issue to be addressed in future investigations. Such work could help to explain the link between environment and AAC in fish eyes.

It is noteworthy that differences in AAC of fish eyes found in this study are in agreement with those reported by Li et al. (2007b) and Li and Ortí (2007) who used the *D. rerio* specie belonging to the Ostariophysi superorder within Teleosts (Lê et al. 1993; Lecointre and Nelson 1996). The most abundant and major vehicle of amino acid delivery in all fish is high-density lipoprotein vitellogenin (Vtg) (Ziv et al. 2008), it’s coded by two major genes Vtga and Vtgb, as well as a minor one, Vtgc. Vtgc is expressed also in Ostariophysi, but at low levels (Wang et al. 2000, 2005). In this context it is important to emphasize that because the Vtg sub domains may be disparately involved in the binding or transporting of nonpolar ligands such as lipids and retinoic acid (Grogan and Taborsky 1987; Sawaguchi et al. 2006). Robust evidences showed that a positive selection of coding genes for proteins is provided by synonymous substitution (Yang and Bielawski 2000), and the change of AA offers a selective advantage.

An interesting concept emerging from the present work is that differences of AAC in fish eyes may provide insight into the different capacity of the animals to adapt to different environmental temperatures. In fact, several authors have defined zebrafish and sardine as eurythermics. Sardines are eurythermic and euryhaline clupeoids that generally inhabit waters with temperatures ranging from 8 to 24 °C and salinities from 30 to 38 psu (Haynes and Nichols 1994; Giannoulaki et al. 2005; Coombs et al. 2006; Petitgas et al. 2006; Stratoudakis et al. 2007; Bonanno et al. 2014). Zebrafish are freshwater fish; even if they are also tolerant to a wide range of salinities that technically extend to brackish conditions. Sawant et al. (2001) found that embryos, reared in salinities of up to 2 parts per thousand, displayed similar rates of survival and hatching in controlled environment at ∼0.3 ppt. They can tolerate a wide temperature range from 6 to 38 °C.

Costas et al. (2012) suggested that acclimation to different environmental temperatures induces several metabolic changes in *Senegalese sole*, suggesting that plasma amino acids (e.g., ASP, GLU and GLY) may be important for thermal acclimation; they showed that temperature affect more drastically concentrations of dispensable amino acids than those of indispensable amino acids and that different exposure temperatures induce different responses. Thus, as in mammals (Liu et al. 2016; Wu et al. 2014; Wu 2014), dietary requirements of all amino acids by fish to meet optimal needs for protein synthesis in tissues (including eyes) are affected by both genotypes and environmental factors. In support of this notion, environmental salinity plays an important role in affecting plasma AAC of fish species (Li et al. 2009). Our results are in agreement with Aragão et al. (2010) who showed that the levels of some indispensable amino acids (HIS, MET and PHE) do not change significantly with environmental salinity, and ILE, LEU and VAL tend to increase with salinity.

## Conclusions

The amino acid composition of the eyes of two fish species (zebrafish and sardine) at the larval stage were determined. The results indicate that eye’s AAC could be used as a useful tool to discriminate the evolutionary origin and species of fish. Although further studies are needed to evaluate the power of such approach, our study showed that the AAC was different between *Sardina pilchardus* and *Danio rerio* species. This does not mean that this technique is sufficient to identify genetic differences between the species, but the data can be used as auxiliary information. Considering that this study stressed the importance of the use of AAC in eyes as a discriminating factor, more experiments are warranted to define the scientific degree of certainty in studies of fish evolution and metabolism.

## Methods

### Zebrafish (*Danio rerio*)

Larvae of zebrafish, which were raised under normal farming conditions, were obtained from Department of Biology, Texas A&M University, College Station, USA, and maintained according to the regulations of the Texas A&M University Animal Care and Use committee. The total number of samples used for the experiment was 11. The fish were used about 4 days of age after hatching, and they were picked up individually to make sure they were still alive. The fish were anesthetized with tricainemethanesulfonate (TMS), also known as MS-222, at the concentration of 200 mg/L in deionized water, with the pH of the solution being adjusted to 7.4 through the addition of sodium bicarbonate. The fish were then fixed in alcohol and the main morphological measurements were taken by means of an optical microscope. The total length of the fish 3.7 mm and the eye diameter was 0.3 mm. Finally, using a pair of needles, the eyes were extracted, dried in an oven at 50 °C to evaporate all the alcohol, and then subjected to acid hydrolysis for the determination of total amino acids.

### Sardine (*Sardina pilchardus*)

Twelve samples of sardine were obtained along the Sicily coast in the Tyrrhenian Sea and used for the experiment, with the approval of the Institute for Coastal and Marine Environment (IAMC), Detached Units of Capo Granitola, Naples, Italy. Fish samples were obtained and preserved in the same manner as described for zebrafish. The total length of the fish (TL) was 33 mm and the eye diameter was 1.6 mm. The fish eyes were extracted and then processed for hydrolysis, as described previously.

### General consideration

Amino acid analysis, although a classical technique, remains indispensable for quality control studies in biochemistry and biotechnology. Over the year, a large number of HPLC methods with fluorescence or UV/visible detection have been developed for the analysis of AAC in protein hydrolysates (Wu et al. 1999; Dai et al. 2014). A successful amino acid analysis depends on the proper performance of the hydrolysis. In fact, other studies have shown that the influence of the hydrolysis conditions represent a major source of error in the analysis (Yüksel et al. 1995). Using our HPLC method, we successfully identified 15 amino acids in fish eyes.

### Method for hydrolysis of protein in fish

The acid hydrolysis method (Dai et al. 2014; Wu et al. 1999) has been used with some modifications. Briefly, two eyes were inserted in a 2-ml glass vial to which was added 1 ml of 6 M HCl. The glass vial was gassed with N_2_ for one min and then capped. All tubes were placed in an oven with 110 °C. Two hours later, the glass vials were gently shacked to ensure that the sample was completely dissolved in the solution. After 20 h of hydrolysis, the glass vials were gently shacked to ensure that the precipitate was suspended in solution. At the end of the 24-h hydrolysis, the whole solution was dried carefully under N_2_. Finally 1 ml of HPLC-grade water was added to each vial and the solution was stored at 4 °C until analyzed within 2 days.

### Amino acid analysis

Amino acids in acid hydrolysates were analyzed with the use of the Waters HPLC apparatus, an analytical column (supelco 3 μm C18 column, 150 mm × 4.6 mm ID) protected by guard column (supelco 5 cm × 4.6 mm), a model 2475 Multi l fluorescence detector and a Millennium-32 workstation (Dai et al. 2014). Fluorescence is monitored at excitation wavelengths of 340 and 455 nm, respectively. The following amino acids were analyzed: aspartate (ASP) plus asparagine (ASN), serine (SER), glutamate (GLU) plus glutamine (GLN), glycine (GLY), histidine (HIS), arginine (ARG), threonine (THR), alanine (ALA), tyrosine (TYR), valine (VAL), lysine (LYS), isoleucine (ILE), leucine (LEU), Phenylalanine (PHE), methionine (MET), cystine (CYS), tryptophane (TRP) and proline (PRO).

### Statistical methods

The unpaired t-test was used to evaluate the significance of observed differences between the two groups of fish. This kind of test belongs to the so-called parametric methods and it is subjected to some assumptions, such as the normality and homoscedasticity. Such properties were checked by means of the Lilliefors and Levene’s test. Even though the assumption of normality and homoscedasticity was met for most AA in both fish species, the same was not verified for several amino acids. As a consequence, we used the Mann–Whitney U test that is the non-parametric analogue of the t-test. The Mann–Whitney U test is conceptually similar to the t-test, except that it is based on the U statistic and does not require normality nor homoscedasticity. Probability values ≤0.05 were taken to indicate statistical significance.

## Abbreviations

AAC: amino acid composition
ASN: aspartate
ASP: asparagine
GLN: glutamine
GLU: glutamate
SER: serine
HIS: histidine
THR: threonine
ARG: arginine
ALA: alanine
TYR: tyrosine
MET: metionine
VAL: valine
PHE: phenylalanine
ILE: isoleucine
LYS: lysine
CYS: cystine
TRP: tryptophane
PRO: proline
GABA: gamma amino butyric acid
